# Movement Disorders and Syndromic Autism: A Systematic Review

**DOI:** 10.1007/s10803-018-3658-y

**Published:** 2018-07-16

**Authors:** L. Bell, A. Wittkowski, D. J. Hare

**Affiliations:** 1 Merseycare NHS Trust, Liverpool, UK; 2 University of Manchester, Manchester, UK; 3 School of Psychology, Cardiff University, 70 Park Place, Cardiff, CF10 3AT UK

**Keywords:** Autism, Retts, Angelman, Movement disorder, Ataxia, Tremor, Dystonia, Rigidity, Extra-pyramidal

## Abstract

Movement disorders are reported in idiopathic autism but the extent to which comparable movement disorders are found in syndromic/co-morbid autism is unknown. A systematic search of Medline, Embase, PsychINFO and CINAHL on the prevalence of specific movement disorder in syndromic autism associated with specific genetic syndromes identified 16 papers, all relating to Angelman syndrome or Rett syndrome. Prevalence rates of 72.7–100% and 25.0–27.3% were reported for ataxia and tremor, respectively, in Angelman syndrome. In Rett syndrome, prevalence rates of 43.6–50% were reported for ataxia and 27.3–48.3% for tremor with additional reports of dystonia, rigidity and pyramidal signs. However, reliable assessment measures were rarely used and recruitment was often not described in sufficient detail.

## Introduction

Movement disorders, such as tremor, ataxia and akinesia, have been shown to have a negative impact on quality of life for adults in the general population (Dodel and Shrag 2010; López-Bastida et al. 2008). For those already limited in terms of their communication and adaptive and social functioning, including individuals with intellectual disabilities (ID) and/or Autism Spectrum Disorder (ASD), a movement disorder is likely to place further restrictions on independence and quality of life. This is clearly demonstrated by first-hand reports highlighting the difficulties experienced by individuals with ASD as a result of movement disorders (e.g., Robledo et al. 2012). Similarly, movement disorders have been frequently identified in individuals with ASD, with ataxia reported in a number of studies (see Fatemi and Folsom 2013), as well as akinesia, dyskinesia, bradykinesia, Tourette syndrome, and catatonic-like symptoms among others (see Donnellan et al. 2013; Breen and Hare 2017), with cerebellum and basal ganglia dysfunction being implicated (see Nayate et al. 2005), resulting in some researchers proposing that ASD could be, at least in part, a disorder of movement (Nayate et al. 2005).

The extent to which comparable movement disorders may be associated with autistic traits *per se* in people with so-called *syndromic* autism is much less clear. Syndromic autism refers to the presence of either diagnosable co-morbid autism or significant autistic traits in people with a distinct genetic developmental disorder, with syndromic autism being particularly prevalent in Angelman (up to 80%), Tuberous Sclerosis Complex (up to 60%), Fragile X (up to 50%), Rett syndrome (40–97% depending on sub-type), CHARGE syndrome (up to 50%) and Down syndrome (up to 39%) (Moss and Howlin 2009; Richards et al. 2015). Moreover, there are clear differences in the specific profile of such autistic traits across different genetic syndromes (e.g., Cochran et al. 2015; Moss et al. 2009, 2013), which may point to different underlying genetic and neurological mechanisms (e.g., Woodcock et al. 2010, 2011).

For some genetic disorders with high rates of syndromic autism, movement disorders form part of the consensus criteria for clinical diagnosis, such as in the case of ataxia in Angelman syndrome (Williams et al. 2006), whilst in others the clinical picture is less clear. To date, the description of movement disorders in such genetic syndromes has traditionally taken the form of case reports (e.g., Fernandez et al. 2000; Holm 1985; Lawson-Yuen et al. 2006; Wright et al. 1992), but this does not permit estimation of prevalence, without which it is not possible to make comparisons across different syndromes, and to examine whether different phenotypic profiles emerge. Similarly, case descriptions have typically been based on clinical observation and judgement (e.g., Bottani et al. 1994; Lawson-Yeun et al. 2006). However, the confidence with which any conclusions can be made regarding the prevalence of movement disorders is dependent in part on the reliability and validity of the assessment method and the overall quality of the research. To date, there has been no systematic review of the extant literature on the prevalence of movement disorders in genetic syndromes with high rates of syndromic autism.

The aims of the current systematic review were to summarise data on the prevalence of movement disorders genetic syndromes with high rates of syndromic autism, to describe the range of assessment methods used in the diagnosis of such movement disorders and to evaluate the quality of research in this area. Selection of syndrome groups for inclusion was predicated on the known rates of syndromic autism (Moss and Howlin 2009; Richards et al. 2015) and on this basis, Angelman syndrome, CHARGE syndrome, Cohen syndrome, Cornelia de Lange syndrome, Fragile X syndrome, Rett syndrome and Tuberous Sclerosis Complex were identified as the basis of the current review.

## Method

The guidelines set out in the Preferred Reporting Items for Systematic Reviews and Meta-Analyses (PRISMA) checklist (Moher et al. 2009) were followed where possible.

### Search Strategy

An electronic literature search was performed by the first author [LH] on 1st April 2016 to identify papers published between 1985 and 2015, across four separate databases: Medline, Embase, Cumulative Index to Nursing and Allied Health Literature (CINAHL) and PsychINFO. Search terms relating to any one of the seven genetic syndromes were combined with terms relating to movement disorders, using the ‘AND’ function. MESH/subject headings were used where possible. Selection of search terms for each of the genetic syndromes was based on the synonyms provided by the National Organisation for Rare Disorders (2016). The selected search terms for movement disorders were intended to identify papers meeting the definition given below and were based in part on an initial search of relevant journals, including *Movement Disorders, Journal of Movement Disorders* and *Journal of Clinical Movement Disorders*, for articles relating to the genetic syndromes of interest. The full list of search terms is provided in Table 1. Searches across each database were limited to journal articles published in the English language that pertained to human participants. The reference lists of relevant papers were examined for other potentially relevant studies not identified through the database search.

**Table 1:** Summary of search terms

Angelman syndrome	‘angelman’ OR ‘happy puppet’ OR Angelman Syndrome (MESH term) OR happy puppet syndrome (MESH term)
CHARGE syndrome	‘charge syndrome’ OR ‘hall-hittner’ OR ‘hall hittner’ OR CHARGE syndrome (MESH term)
Cohen syndrome	‘cohen’ OR ‘pepper syndrome’ OR Cohen Syndrome (MESH term)
Cornelia de Lange syndrome	‘cornelia de lange’ OR ‘cornelia-de-lange’ OR ‘brachmann de lange’ OR ‘brachmann-de-lange’ OR ‘cdls’ OR ‘bdls’ OR ‘de lange’ OR ‘de-lange’ OR ‘amsterdam syndrome’ OR Cornelia de Lange Syndrome (MESH term) OR De Lange Syndrome (MESH term)
Fragile X syndrome	‘fragile x’ OR ‘fragile-x’ OR ‘fragile site’ OR ‘fxs’ OR ‘fra(X)’ OR ‘fraX’ OR ‘FMRP’ OR ‘marker x’ OR ‘martin-bell’ OR ‘martin bell’ OR ‘x-linked mental retardation’ or Fragile X Syndrome (MESH term) OR Mental Retardation, X-Linked (MESH term)
Rett syndrome	‘rett’ OR ‘rtt’ OR Rett Syndrome (MESH term)
Tuberous sclerosis complex	‘tuberous sclerosis’ OR ‘tuberose sclerosis’ OR ‘TSC’ OR ‘phakomatosis ts’ OR ‘bourneville pringle’ OR Tuberous Sclerosis (MESH term)
Movement disorder	‘movement’ OR ‘motor’ OR ‘movement disorder*’ OR ‘ataxi*’ OR ‘apraxi*’ OR ‘gait’ OR ‘tremor’ OR ‘parkinson*’ OR ‘dyskinesia’ OR ‘akinesia’ OR ‘cataton*’ OR Movement (MESH term) OR Motor Skills Disorder (MESH term) OR Movement Disorders (MESH term) OR Ataxia (MESH term) OR Apraxias (MESH term) OR Gait (MESH term) OR Gait Disorders (MESH term) OR Tremor (MESH term) OR Parkinsonian Disorders (MESH term) OR Dyskinesias (MESH term) OR Catatonia (MESH term)

### Inclusion and Exclusion Criteria

Studies were required to meet the following inclusion criteria:

1. Written in the English language
2. Published as a full length report in a peer-reviewed journal
3. Provide a clear description regarding the recruitment/selection of participants
4. Provide information regarding the age/age range of participants
5. Specify individuals with Angelman syndrome, CHARGE syndrome, Cohen syndrome, Cornelia de Lange syndrome, Fragile X syndrome, Rett syndrome or Tuberous Sclerosis Complex syndrome as the main population being researched
6. Include participants whose genetic syndrome has been confirmed through DNA analysis
7. Provide a description of the assessment tool or procedure used
8. Report on identified movement disorder, defined as *a neurological condition resulting in abnormal or slowed movement*.

If papers included participants both with and without genetic confirmation of the syndrome of interest, they were only included if data were presented separately for those with and without genetic confirmation by DNA analysis. Studies were excluded if they did not constitute a stand-alone paper/full length report (e.g., letters to the editor, commentaries, published conference abstracts), if they related to developmental motor skills or to biomechanical aspects of movement (e.g., scoliosis or hypotonia), if participants were selected on the basis of having an identified movement disorder, or if they provided insufficient detail to determine eligibility for inclusion. Studies reporting on stereotyped movements were not included for the purposes of the current review, as the prevalence of repetitive and stereotyped behaviours in genetic syndromes has been described elsewhere (e.g., Moss et al. 2009), and stereotypy may sometimes be considered a functional behaviour (Cunningham and Schreibman 2008).

### Data Extraction

Data relating to the prevalence of any specified movement disorder were extracted from included papers. Authors, year of publication, sample size and characteristics, recruitment method, genetic mechanism and method of assessment were also recorded.

### Quality Assessment

Relatively few quality rating tools are available for evaluating prevalence studies, and there is wide variability in the quality of those tools which are available (see Sanderson et al. 2007). For the current review the risk of bias assessment developed by LeBoeuf-Yde and Lauritsen (1995) and later revised by Hoy et al. (2012) was used. Hoy et al. (2012) reported good inter-rater reliability for their Risk of Bias assessment as well as positive feedback regarding ease of use. Studies are rated as ‘High’ versus ‘Low’ risk of bias across ten domains, which relate to the internal and external validity of the findings. An overall summary rating of ‘Low’, ‘Moderate’ or ‘High’ risk is given, based on responses across the ten items. For the current review a second independent rater scored 50% of papers. A Kappa of 0.53 was obtained for the overall risk of bias rating, indicating fair reliability (Fleiss 1981).

## Results

**Fig. 1:**
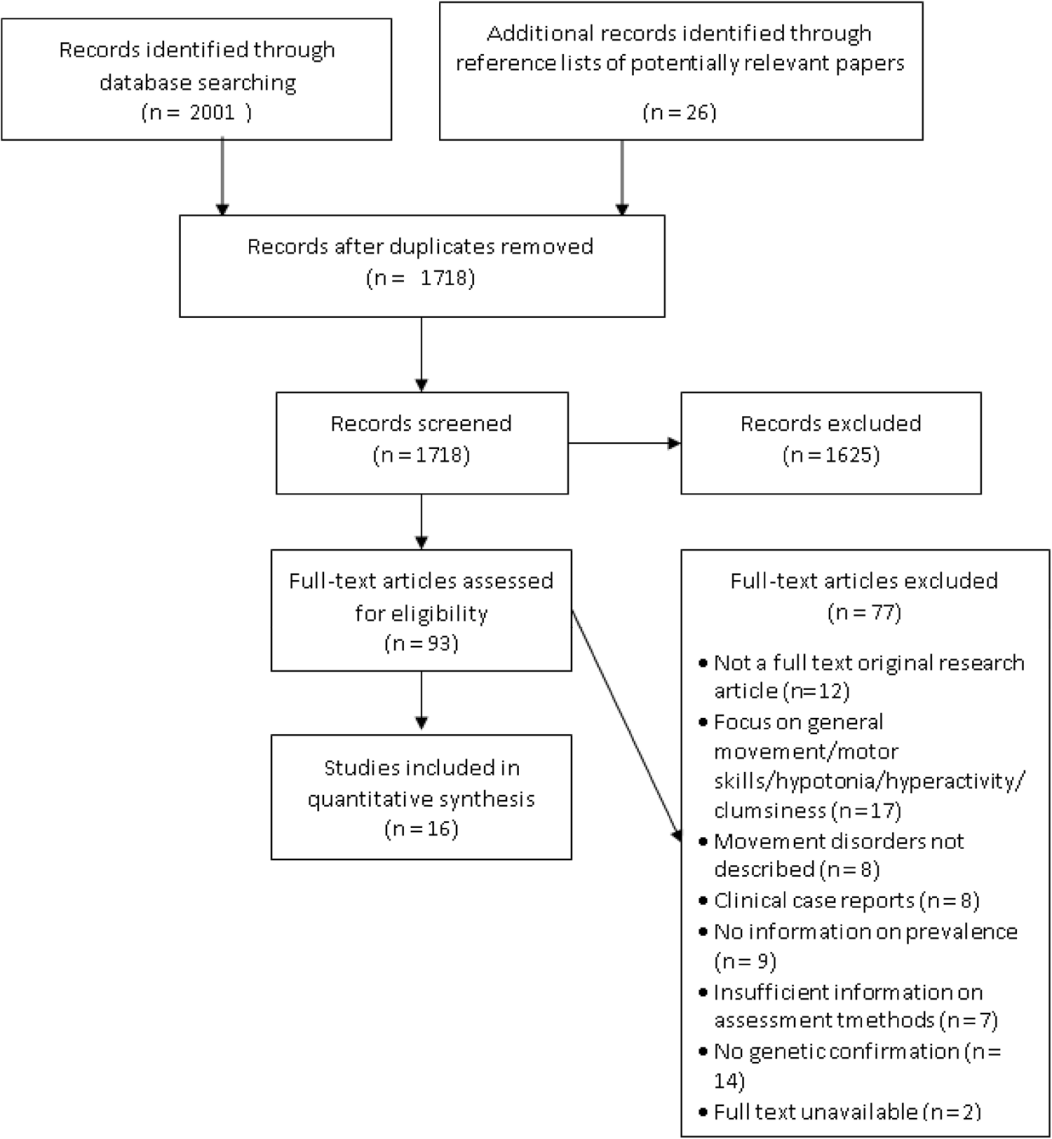
PRISMA diagram for selection of papers

The initial database search returned a total of 2001 papers, of which 309 were duplicates. After screening titles and abstracts 83 potentially relevant papers were identified, with a further ten papers identified through the references lists of these 83 papers. Of these 93 papers 16 were identified as meeting the criteria for inclusion in the review. The PRISMA diagram (Moher et al. 2009) presented in Fig. 1 provides a summary of the search results, including the reasons for exclusion of papers. All of the 16 papers meeting criteria for inclusion related to individuals with either Angelman syndrome or Rett syndrome. Of all 16 studies identified, only three focused explicitly on disorders of movement. In the remaining studies movement disorders were reported as part of a broader description of clinical characteristics. Results are reported separately by syndrome, and an overview of common methodological issues is provided. A summary of all 16 papers is provided in Table 2 and a full breakdown of quality ratings for each paper is provided in Table 3.

**Table 2:** Sample characteristics, methodology, and quality assessment for studies reporting the prevalence of movement disorders in Angelman syndrome and Rett syndrome

Authors	Syndrome	N	% Male	Age range *(M, SD)*	Genetic mechanism	Recruitment information	Assessment method	Movement disorders identified	% Prev. (*N*)	Risk of bias
Bai et al. (2014)	Angelman	30	50.0	2.7–9.6 years at last observation (M 5.3 years, SD 2.1 years)	Deletion	Sample of children with Angelman syndrome in China—recruitment method not specified	Purpose-made questionnaire based on clinical criteria	Ataxic movement	100% (24) of those able to walk	Moderate
Beckung et al. (2004)	Angelman	11	Unclear for subgroup with DNA confirmation	Unclear for subgroup with DNA confirmation	Not reported	Individuals referred to a Children’s Hospital in Sweden for investigation of Angelman syndrome	Extensive clinical investigation—movement problems classified based on performance	Gait ataxia Tremor	72.7% (8) 27.3% (3)	Moderate
Buoni et al. (1999)	Angelman	11	54.5	1 year 6 months to 15 years at last observation (Not reported)	10 deletion 1 UPD	Genetic screening of 144 patients at Paediatric Institute	Medical records, history and clinical examination	Ataxia	100% (11)	Moderate
Clayton-Smith (2001)	Angelman	28	42.9	16–40 years (Not reported)	19 deletion 7 UBE3A mutation 1UPD 1 imprinting defect	Participated in a previous study or seen personally by the author	Medical notes, parent/carer-reported history and clinical examination	Ataxic gait Worsening Tremor	100% (28) 25.0% (7)	Moderate
Guerrini et al. (1996)	Angelman	11	45.5	3–28 years (Not reported)	8 deletion 2 UPD 1 microdeletion	Unclear	Rated scale score based on observation	Jerky, tremulous, dystonic movement	100% (11)	Moderate
Moncla et al. (1999)	Angelman	14	57.1	9 months–33 years at diagnosis (Not reported)	All UBE3A mutation	Referred to the authors in Department of Genetic Medicine	Clinical history, anthropometric data and physical and neurological findings	Ataxia	100% (14) total 21.4% (3) typical 21.4% (3) mild 57.1% (8) extremely mild	Moderate
Saitoh et al. (1994)	Angelman	40	47.5	Unclear	37 deletion 3 sub-microscopic deletion	Large sample of individuals with Angelman syndrome in Japan – recruitment method not specified	Questionnaires completed by physicians	Ataxic movement	97.1% (34) of those for whom ataxic movement data reported	High
Sandanam et al. (1997)	Angelman	11	81.8	24–36 years at last review (M 31.5 years)	All deletion	Genetic screening of residents of two residential institutions	Review of records, history and clinical examination completed by two clinicians	Ataxic gait	100% (9) of those able to walk	Moderate
Smith et al. (1996)	Angelman	27	33.3	3–34 years at last review (M 11.2 years)	Deletion	Referrals for genetic testing from physicians across Australia and New Zealand as part of a research grant	Data sheet based on clinical criteria plus medical records, parent interviews, photos and videos where available, and either correspondence with referrer or clinical examination by authors	Ataxia—wide-based gait, unsteadiness with jerky movements or thumping heavy gait	100% (27)	Moderate
Smith et al. (1997)	Angelman	4	50.0	7–11 years (M 8 years)	Uniparental disomy	As above	Data sheet based on clinical criteria, plus medical records, parent interviews and correspondence with referrer	Ataxia	100% (4) total 75% (3) mild and most evident when excited	High
Tan et al. (2011)	Angelman	92	54.3	5–60 months (Median 33.5 months)	68 deletion 13 UPD/imprinting 11 UBE3A mutation	Part of a multi-centre study with enrolment through support groups and referral from professionals	Structured medical history and physical examination by a clinical geneticist	Ataxic gait	87.8% (36) of those able to walk 95.5% (21) deletion 72.7% (8) UPD/imprinting 87.5%(7) UBE3A	Moderate
Zori et al. (1992)	Angelman	16	Unclear	Unclear—children	Deletion	American families with a child with Angelman syndrome —recruitment method not specified for these families	Family questionnaire assessing clinical, cytogenetic and developmental variables, plus physical examination and lab data	Ataxic gait Jerky gait	81.3% (13) 93.8% (15)	Moderate
Bartholdi et al. (2006)	Rett	4	0	4–19 years at last observation (Not reported)	Exon 1 mutation/genomic rearrangement in MECP2	Recruited from a larger cohort of individuals with Rett syndrome in Switzerland. No further details	Medical records and clinical examination	Gait ataxia	25,% (1), 50% of those walking	Moderate
Einspieler et al. (2005)	Rett	12	0	0–6 months (Not reported)	6 truncating mutations, 3 missence mutations, 2 deletions, 1 substitution at 401	Videotapes donated by British families to the project	Videotapes analysed by two observers	Tremor Abnormal general movement	27.3% (3) 100% (9)	Moderate
Smeets et al. (2005)	Rett	10	0	3–54 years at diagnosis (Not reported)	Hotspot deletion in C-terminal segment of MECP2	Seen by first author clinically—Clinical Genetics Department in a University Hospital in Belgium	Observed and assessed by first author and parents/carers	‘Awkward’ walking pattern Short stiff steps as seen in Parkinson’s disease	20.0% (2) 10.0% (1)	Moderate
Temudo et al. (2008)	Rett	60	Not reported. Not all female	5–13.5 years (Median 7.0)	26 missence mutations, 34 truncating mutations	Referred to the project by any Paediatric Neurologist across Portugal	Motor-Behavioral Assessment Scale for Rett syndrome	Ataxia	35.0% (21) 46.2% (12) Missence (M) 26.5% (9) Truncating (T)	Moderate
								Ataxic/rigid gait	43.6% (unclear) 36.8% M 50.0% T	
								Dystonia	63.3% (38) 46.2% (12) M 76.5% (26) T	
								Rigidity	48.3% (29) 34.6% (10) M 58.8% (20) T	
								Pyramidal signs	28.3% (17) 23.1% (6) M 32.4% (11) T	
								Tremor	48.3% (29) 50.0% (13) M 47.1% (16) T	

**Table 3:** Summary of risk of bias ratings by paper

Author	Sample represents national population	Sample represents target population	Random selection	Non-response bias	Direct measure	Case definition given	Reliable and valid measure	Same data collected from all	Appropriate prevalence period	Numerator/denominator appropriate	Summary rating
Angelman syndrome papers											
Bai et al. (2014)	High	High	High	High	High	High	High	Low	Low	Low	Moderate
Beckung et al. (2004)	High	High	High	High	Low	Low	High	Low	Low	Low	Moderate
Buoni et al. (1999)	High	High	High	High	Low	High	High	Low	Low	Low	Moderate
Clayton-Smith (2001)	High	High	High	High	High	High	High	Low	Low	Low	Moderate
Guerrini et al. (1996)	High	High	High	High	Low	Low	High	Low	Low	Low	Moderate
Moncla et al. (1999)	High	High	High	High	Low	High	High	Low	Low	Low	Moderate
Saitoh et al. (1994)	High	High	High	High	High	High	High	Low	High	High	High
Sandanam et al. (1997)	High	High	High	Low	Low	High	High	Low	Low	Low	Moderate
Smith et al. (1996)	Low	High	High	High	High	Low	High	High	High	Low	Moderate
Smith et al. (1997)	Low	High	High	High	High	High	High	Low	High	High	High
Tan et al. (2011)	High	Low	Low	High	Low	High	High	Low	Low	Low	Moderate
Zori et al. (1992)	Low	High	High	High	Low	High	High	Low	Low	Low	Moderate

Author	Item 1	Item 2	Item 3	Item 4	Item 5	Item 6	Item 7	Item 8	Item 9	Item 10	Summary rating
Rett syndrome papers											
Bartholdi et al. (2006)	High	High	High	High	Low	High	High	Low	Low	Low	Moderate
Einspieler et al. (2005)	High	High	High	High	Low	Low	Low	Low	Low	Low	Moderate
Smeets et al. (2005)	High	High	High	High	Low	Low	High	Low	Low	Low	Moderate
Temudo et al. (2008)	Low	Low	Low	High	Low	High	High	Low	Low	High	Moderate

### Movement Disorder in Angelman Syndrome

Out of the 16 papers identified, 12 related to movement disorder in Angelman syndrome and could be broadly categorised into those based on clinical assessment (clinical examination, medical records and/or history taking) and those utilising idiosyncratic questionnaires. Several papers reported clinical characteristics across larger cohorts of individuals, some of whom were diagnosed based only on clinical criteria (Beckung et al. 2004; Buoni et al. 1999; Saitoh et al. 1994; Zori et al. 1992). In each case, results are only reported for the sub-group of individuals with genetic confirmation of Angelman syndrome.

Papers examining movement disorders in Angelman syndrome were focused almost exclusively on the presence of ataxic/jerky movements, considered phenotypic of Angelman syndrome (Williams et al. 2006), with three exceptions (Beckung et al. 2004; Clayton-Smith 2001; Guerrini et al. 1996). The number of participants included in the studies ranged from 4 to 92, due in part to the underlying genetic mechanism, with the smallest sample (Smith et al. 1997) being made up exclusively of individuals with uniparental disomy (UPD), a less common mechanism in Angelman syndrome than maternal deletion (see Williams et al. 2010). The 12 papers covered a broad range of ages, with five studies focused exclusively on children (Bai et al. 2014; Buoni et al. 1999; Smith et al. 1997; Tan et al. 2011; Zori et al. 1992), two exclusively on adults (Clayton-Smith 2001; Sandanam et al. 1997), and three focused on children and adults (Guerrini et al. 1996; Moncla et al. 1999; Smith et al. 1996). For the remaining two studies (Beckung et al. 2004; Saitoh et al. 1994) it was not possible to determine the age of the subgroup of individuals with genetically confirmed Angelman syndrome.

The risk of bias was variable across studies. Whilst the majority were considered to be at moderate risk of bias, Saitoh et al.’s (1994) and Smith et al.’s (1996) papers were both judged to be at high risk of bias. None of the 12 papers reported on the validity or reliability of the assessment method for movement disorders. Whilst clinical assessment may be considered most analogous to the means by which movement disorders are typically diagnosed in medical practice, none of the studies report evidence to support the reliability or validity of this method. One paper (Sandanam et al. 1997) did report using at least two clinicians for each assessment, which might be expected to increase the reliability of the clinical judgements made, but it was not possible to determine whether this was the case, and inter-rater reliability information was not provided. Whilst the use of a questionnaire or data sheet might ensure consistency in the information asked of each informant, the likely accuracy of this information was unclear, as none of the studies using questionnaire methods (Bai et al. 2014; Saitoh et al. 1994; Smith et al. 1996, 1997; Zori et al. 1992) reported on the psychometric properties of their purpose-made measures.

The reported prevalence of ataxic/jerky movements in Angelman syndrome ranged from 72.7% (Beckung et al. 2004; Tan et al. 2011) to 100% (Bai et al. 2014; Buoni et al. 1999; Clayton-Smith 2001; Moncla et al. 1999; Sandanam et al. 1997; Smith et al. 1996, 1997), across the 11 studies that provided prevalence estimates. In those with a non-deletion mechanism, ataxic/jerky movement was often reported to be milder or less prevalent (e.g., Moncla et al. 1999; Tan et al. 2011; Smith et al. 1997). Two studies also reported on the prevalence of tremor in individuals with Angelman syndrome. The prevalence estimates of 27.3% (Beckung et al. 2004) and 25.0% (Clayton-Smith 2001) appeared relatively consistent and indicated that around a quarter of individuals were affected by tremor, although Clayton-Smith did not elaborate on what is meant by ‘worsening tremor’. Finally, one study reported that all participants presented with a jerky, tremulous, dystonic movement, which was determined to be a result of cortical myoclonus (Guerrini et al. 1996).

### Movement Disorder in Rett Syndrome

Four papers relating to Rett Syndrome were identified and are summarised in Table 2. One of these papers (Einspieler et al. 2005) included participants with and without genetically confirmed Rett syndrome, therefore calculated prevalence rates were based only on those with an identified genetic mechanism. All studies were published in the last 11 years, which is probably due to the relatively recent progress in understanding the genetic basis of Rett syndrome (Amir et al. 1999), which was only fully defined clinically in the 1980s (Hagberg et al. 1985). The sample size of studies varied between 4 and 60 participants and, again, this was partly dependent on the underlying genetic mechanism. Bartholdi et al.’s (2006) study consisted of four girls with Rett syndrome, but the focus of their report was on describing the clinical findings in individuals with relatively uncommon underlying mechanisms, namely exon 1 mutations and genomic rearrangements in MECP2. Three of the four studies focused on children and adolescents (Bartholdi et al. 2006; Einspieler et al. 2005; Temudo et al. 2008). Only one adult was identified (Smeets et al. 2005), which may reflect greater interest in the characteristic regression period that begins in childhood (see Hagberg 2002; Neul et al. 2010).

Recruitment methods differed across each of the studies, including self-selection (Einspieler et al. 2005), referral by relevant clinicians (Temudo et al. 2008), retrospective study of individuals seen by the author in clinical practice (Smeets et al. 2005) and recruitment of individuals from an existing cohort (Bartholdi et al. 2006). Similarly, the methods of assessment used varied, with two studies (Bartholdi et al. 2006; Smeets et al. 2005) reliant on clinical examination and/or medical records whilst the other researchers developed idiosyncratic assessment methods for the identification of movement disorders. Temudo et al. (2008) did not report the psychometric properties of their Motor-Behavioral Assessment Scale, whilst Einspieler et al. (2005) reported 94% agreement between raters and 92% test–retest agreement.

The risk of bias was moderate across each of the studies, with Bartholdi et al. (2006) study methods and reporting being the most susceptible to bias. Across each of the studies, particular concerns were identified in relation to the lack of information on the representativeness of the sample and possible selection bias. However, relative strengths of each of the studies were that the same mode of data collection was applied for all participants, and participants were directly observed by the authors.

It was possible to obtain a prevalence estimate for seven different aspects of disordered movement within Rett syndrome. Two studies provided information on ataxic gait (Bartholdi et al. 2006; Temudo et al. 2008), with prevalence estimates relatively consistent despite different underlying genetic mechanisms. Between 43.6 and 50.0% of those able to walk were reported to show ataxic gait. Tremor was also examined across two separate studies, but the estimated prevalence varied from 27.3% in young infants (Einspieler et al. 2005) to 48.3% in older children (Temudo et al. 2008). Some form of abnormal general movement was recorded in 100% of 0–6-month-olds observed by Einspieler and colleagues. They describe this as an absence of normal fidgety movements, jerky or abnormally slow movements, or abrupt and disorganised movements, present in 30, 35 and 35% of children, respectively.

In Temudo et al.’s (2008) study, various additional movement problems were identified. Ataxia was reported in 35.0% of children, dystonia in 63.3%, rigidity in 48.3% and pyramidal signs in 28.3%. There were some apparent differences in prevalence dependent on underlying genetic mechanisms, with ataxia reported in 46.2 and 26.5% of individuals with missense and truncating mutations, respectively, dystonia reported in 46.2 and 76.5%, respectively, rigidity reported in 34.6 and 58.8%, respectively, pyramidal signs reported in 23.1 and 32.4%, respectively, and ataxic gait reported in 36.8 and 50.0%, respectively. However, Temudo and colleagues did not report on the significance of these group differences.

### Common Methodological Issues

There were common issues in all 16 of the papers included in the current review. Regarding external validity of the research, nine of the studies did not describe recruitment methods in sufficient detail to allow judgements to be made regarding the representativeness of the sampling frame, possible selection bias within the sampling frame, or the likelihood of response bias (Bai et al. 2014; Bartholdi et al. 2006; Buoni et al. 1999; Clayton-Smith 2001; Guerrini et al. 1996; Moncla et al. 1999; Saitoh et al. 1994; Smeets et al. 2005; Zori et al. 1992). Whilst two studies referred to recruitment from a larger cohort or participants of a previous study, the recruitment of participants into these original cohorts was not described. A further three studies relied on a sampling frame that may not have been representative of the target population and did not use random selection or consensus sampling within their sampling frame (Beckung et al. 2004; Einspieler et al. 2005; Sandanam et al. 1997). For example, Sandanam and colleagues recruited their sample through institutional settings only and did not genetically screen all of the residents for possible Angelman syndrome. Issues of possible response bias were rarely discussed, possibly because information was collected through routine clinical assessment in many cases.

Regarding internal validity, only five studies provided any definition for the movement disorder in question (Beckung et al. 2004; Einspieler et al. 2005; Guerrini et al. 1996; Smeets et al. 2005; Smith et al. 1996) and the reliability of the assessment method was reported in only one study (Einspieler et al. 2005). In four studies (Bai et al. 2014; Saitoh et al. 1994; Smith et al. 1996, 1997) no direct observation of the participants was conducted by the authors, increasing the risk of bias in these estimates. In one further study (Clayton-Smith 2001), it was unclear whether the entire sample had been examined clinically by the author.

In terms of the relative strengths of the research under review, all but one paper (Smith et al. 1996) applied the same methods to each of the study participants, and with two exceptions (Saitoh et al. 1994; Temudo et al. 2008), all of the studies provided an appropriate numerator and denominator for the calculation of prevalence.

## Discussion

This was the first paper to systematically review and evaluate research on the prevalence of movement disorders in genetic syndromes with high rates of syndromic autism and to report on the range of assessment methods used for the identification of movement disorders within this population. In addition, the current review adds to our overall understanding of the behavioural phenotypes of Angelman and Rett syndromes as well as highlighting important gaps in the literature. Although the review was designed to focus on movement disorders in those syndromes with high rates of syndromic autism, namely Angelman syndrome, CHARGE syndrome, Cohen syndrome, Cornelia de Lange syndrome, Fragile X syndrome, Rett syndrome and Tuberous Sclerosis Complex, of the 16 studies meeting the inclusion criteria, twelve related to Angelman syndrome and four to Rett syndrome. Studies relating to any of the other syndromes did not met the criteria for inclusion. The apparent over-representation of Angelman and Rett syndrome among the results of the review may reflect the fact that movement disorders are considered part of the diagnostic criteria in these groups (Neul et al. 2010; Williams et al. 2006). As should always be the case for a systematic reviews, the specific search terms and criteria were developed in advance of performing the literature search, and therefore it was not anticipated that only papers relating to Angelman and Rett syndromes would meet the inclusion criteria for the formal review.

Assessment methods included clinical assessment (clinical examination, medical records and/or clinical history taking), questionnaires and assessment scales, and a video observation method used in one study (Einspieler et al. 2005). The majority of studies in relation to Angelman syndrome focused on ataxia or ataxic/jerky gait. Perhaps unsurprisingly, given that ataxia is considered to be a diagnostic characteristic of Angelman syndrome (Williams et al. 2006), a number of studies reported a 100% prevalence rate, with the lowest reported prevalence being 72.7% in those with uniparental disomy (UPD) or an unspecified underlying genetic mechanism (Beckung et al. 2004; Tan et al. 2011). Tremor was reported in around a quarter of individuals with Angelman syndrome (Beckung et al. 2004; Clayton-Smith 2001). Ataxic movements were reported less commonly in Rett syndrome, affecting 50% or fewer individuals (Bartholdi et al. 2006; Temudo et al. 2008). Other movement problems were reported in Rett syndrome, including dystonia in 63.3% and pyramidal signs in 28.3% (Temudo et al. 2008). The prevalence of tremor was estimated at 27.3% in young children (Einspieler et al. 2005), similar to the prevalence in Angelman syndrome, although for older children this rose to 48.3% (Temudo et al. 2008). Across the majority of studies, moderate levels of bias were introduced into the findings, due to issues such as lack of detail in reporting recruitment methods, absence of reliability and validity information for the assessment methods used, and failure to provide a definition for the movement disorders reported.

The conclusions that can be reached about movement disorders in genetic syndromes associated with syndromic autism are inevitably limited by the lack of available cohort studies in relation to CHARGE syndrome, Cohen syndrome, Cornelia de Lange syndrome, Fragile X syndrome and Tuberous Sclerosis Complex, as well as the level of methodological bias identified in those studies reporting on the prevalence of movement disorders in Angelman and Rett syndromes. In order to increase the level of confidence in reported prevalence data, future research should focus on developing valid and reliable assessment tools for identifying movement disorders in these groups, based on standardised definitions. Authors must also ensure that recruitment methods are reported in sufficient detail.

In terms of the review strategy presented here, it could be argued that the requirement for an underlying genetic mechanism to be identified would have led to the exclusion of a number of potentially relevant studies, as in some cases advances in our understanding of the underlying mechanisms have occurred only relatively recently (e.g., Amir et al. 1999; Kolehmainen et al. 2003; Krantz et al. 2004). All but one of the papers excluded based on the absence of DNA confirmation of the genetic syndrome related to Angelman or Rett syndrome. The other study excluded on this basis related to a small cohort of children with Cornelia de Lange syndrome. In their sample of five children, Leroy et al. (1993) reported a wide-based unsteady gait in two out of the three children able to walk. Although the criterion for genetic confirmation led to the exclusion of a number of papers, it was deemed necessary to reduce the bias introduced by examining the prevalence of specific clinical characteristics in a clinically defined sample. This appears particularly important, given that 11 out of 12 studies in Angelman syndrome focused primarily on ataxic/jerky gait, which is one of the criteria for clinical diagnosis (Williams et al. 2006). Similarly, case reports were excluded from this review. Whilst they do not contribute to estimates of prevalence, such papers can provide valuable insights into movement disorders in individuals with genetic syndromes. For example, one notable paper described presentations of Gilles de la Tourette syndrome and tic disorder in five individuals with Fragile X syndrome (Schneider et al. 2008). Other case reports have described issues such as chorea in a woman with Tuberous Sclerosis Complex (Wright et al. 1992) and Parkinsonian symptoms in a man with probable Cornelia de Lange syndrome (Fernandez et al. 2000).

Although it is possible that the selected search terms did not capture each specific movement disorder that has been described in the population of interest, due to the vast number of possible movement disorders, it is also likely that the broader search terms such as ‘movement disorder’ captured the majority of relevant papers. For example, papers in relation to tics (Schneider et al. 2008) and extrapyramidal signs (Fitzgerald et al. 1990; Temudo et al. 2008) were identified, despite the fact that search terms specific to these disorders were not used.

Finally, the quality assessment tool selected for this review was found to have only ‘fair’ inter-rater reliability (Kappa = 0.53). This may be a reflection on the applicability of the tool to research in this area. It may also highlight a general lack of clear unambiguous reporting within this literature, leading to difficulties in drawing confident conclusions about the level of bias.

### Implications and Future Research

The results of this review indicate that the majority of individuals with Angelman syndrome and Rett syndrome experience some form of movement disorder, with prevalence of specific movement disorders varying across syndrome, with ataxic/jerky gait being more prevalent in Angelman syndrome than Rett syndrome, and a conversely greater proportion of people with Rett syndrome experiencing tremor, at least in older children. In line with findings in relation to idiopathic autism (Fatemi and Folsom 2013), many individuals with either Angelman or and syndrome exhibited ataxic gait, indicating that the risk of specific movement disorders may be higher in individuals with other features of ASD, although the finding of higher rates of ataxia in Angelman syndrome despite a lower prevalence of syndromic autism (Richards et al. 2015) indicates that other, possibly syndrome-specific, factors are likely to be involved. There is, therefore, an urgent need for research to examine the relationship, if any, between specific movement disorders and autism symptomatology in at least these two syndromes associated with high rates of syndromic autism.

As well as causing possible discomfort and distress, issues such as ataxia, tremor, dystonia and other involuntary movements are likely to pose serious limitations on the ability of people with Angelman and Rett syndrome engage in a variety of activities without significant support. Such difficulties add to the complex picture of support needs in people with Angelman and Rett syndrome, who are also likely to be non-verbal (Jolleff and Ryan 1993; Neul et al. 2010; Penner et al. 1993) and may display behaviours that risk harm to themselves or those around them (e.g., Arron et al. 2011; Hagberg et al. 1983), and are likely to contribute to the higher levels of stress experienced by parents (Griffith et al. 2011; Laurvick et al. 2006; Perry et al. 1992). At a service level, within the field of clinical psychology the majority of neuropsychological assessments used in standard clinical practice require a reasonable level of motor control in the execution of responses, even in the case of those developed explicitly for individuals with severe intellectual disabilities (e.g., Albert and Cohen 1992; Wechsler and Naglieri 2006). Services and care providers must make appropriate adjustments to the physical and social environment to accommodate the needs of children and adults with Angelman and Rett syndrome.

The overall lack of research into the prevalence of movement disorders in the majority of genetic syndromes investigated here may relate to the wider issue of diagnostic overshadowing (Reiss et al. 1982), whereby movement problems are seen as part and parcel of having either ASD or an intellectual disability, with clinicians and researchers alike failing to investigate and identify specific disorders of movement that contribute to the wider clinical profile. This is illustrated by Leary and Hill (1996), who found evidence indicating that the same movement problems that might be attributed to neurological disorder in other individuals are more likely to be perceived as behavioural in people with ASD. Future research should build on clinical case reports to examine the prevalence of identified movement disorders within larger cohorts of individuals with a specific genetic syndrome. Consideration should also be given to possible differences according to the underlying genetic mechanism.

## Conclusions

Sixteen papers met the criteria for inclusion within the current systematic literature review and related to the prevalence of movement disorders in either Angelman syndrome and Rett syndrome, both of which are strongly associated with ASD. The review highlighted that the majority of individuals with both Angelman and Rett syndrome were affected by some form of movement disorder, with up to 100% of people with Angelman syndrome displaying ataxia, and the majority of those with Rett syndrome exhibiting dystonia. However, issues with methodological bias were consistently identified across studies. These findings highlight the need for services to acknowledge and assess for movement disorders when supporting individuals with Angelman and Rett syndromes. The risks posed to the person’s physical and emotional wellbeing must be considered and services must work to reduce the impact of movement disorders on the lives of people and their families. Future research should focus on expanding on the current literature, by examining movement disorders in other genetic syndromes, and by employing more robust methods of assessment.
